# A Digital Parenting Intervention With Intimate Partner Violence Prevention Content: Quantitative Pre-Post Pilot Study

**DOI:** 10.2196/58611

**Published:** 2025-01-03

**Authors:** Moa Schafer, Jamie Lachman, Paula Zinser, Francisco Antonio Calderón Alfaro, Qing Han, Chiara Facciola, Lily Clements, Frances Gardner, Genevieve Haupt Ronnie, Ross Sheil

**Affiliations:** 1 Centre for Evidence Based Intervention Department of Social Policy and Intervention University of Oxford Oxford United Kingdom; 2 Centre for Social Science Research University of Cape Town Cape Town South Africa; 3 Innovations in Development, Education, and the Mathematical Sciences (IDEMS) International Reading United Kingdom; 4 UNICEF Jamaica Kingston Jamaica

**Keywords:** intimate partner violence, SMS text messaging, chatbot, user engagement, parenting, violence, mobile phone

## Abstract

**Background:**

Intimate partner violence (IPV) and violence against children are global issues with severe consequences. Intersections shared by the 2 forms of violence have led to calls for joint programming efforts to prevent both IPV and violence against children. Parenting programs have been identified as a key entry point for addressing multiple forms of family violence. Building on the IPV prevention material that has been integrated into the parenting program ParentText, a digital parenting chatbot, this pilot study seeks to explore parents’ engagement with the IPV prevention content in ParentText and explore preliminary changes in IPV.

**Objective:**

This study aimed to assess parents’ and caregivers’ level of engagement with the IPV prevention material in the ParentText chatbot and explore preliminary changes in experiences and perpetration of IPV, attitudes toward IPV, and gender-equitable behaviors following the intervention.

**Methods:**

Caregivers of children aged between 0 and 18 years were recruited through convenience sampling by research assistants in Cape Town, South Africa, and by UNICEF (United Nations Children's Fund) Jamaica staff in 3 parishes of Jamaica. Quantitative data from women in Jamaica (n=28) and South Africa (n=19) and men in South Africa (n=21) were collected electronically via weblinks sent to caregivers’ phones using Open Data Kit. The primary outcome was IPV experience (women) and perpetration (men), with secondary outcomes including gender-equitable behaviors and attitudes toward IPV. Descriptive statistics were used to report sociodemographic characteristics and engagement outcomes. Chi-square tests and 2-tailed paired dependent-sample *t* tests were used to investigate potential changes in IPV outcomes between pretest and posttest.

**Results:**

The average daily interaction rate with the program was 0.57 and 0.59 interactions per day for women and men in South Africa, and 0.21 for women in Jamaica. The rate of completion of at least 1 IPV prevention topic was 25% (5/20) for women and 5% (1/20) for men in South Africa, and 21% (6/28) for women in Jamaica. Exploratory analyses indicated significant pre-post reductions in overall IPV experience among women in South Africa (*P=*.01) and Jamaica (*P*=.01) and in men’s overall harmful IPV attitudes (*P*=.01) and increases in men’s overall gender-equitable behaviors (*P*=.02) in South Africa.

**Conclusions:**

To the best of our knowledge, this is the first pilot study to investigate user engagement with and indicative outcomes of a digital parenting intervention with integrated IPV prevention content. Study findings provide valuable insights into user interactions with the chatbot and shed light on challenges related to low levels of chatbot engagement. Indicative results suggest promising yet modest reductions in IPV and improvements in attitudes after the program. Further research using a randomized controlled trial is warranted to establish causality.

## Introduction

### Background

Violence against women (VAW) and violence against children (VAC) are global issues with severe, long-lasting consequences, which affect individuals and communities worldwide. Global reports have revealed alarmingly high rates of both forms of violence [1,2]. Prevalence estimates of intimate partner violence (IPV), which is the most common form of VAW, have found that >27% of ever-partnered women aged >15 years have experienced physical IPV, sexual IPV, or both at least once in their life [1]. Reports of VAC are also of great concern, with systematic review findings suggesting that >50% of all children worldwide, that is, >1 billion children globally, have experienced past-year violence [3,4]. Research findings also indicate that prevalence rates are higher for both forms of violence in low- and middle-income countries [5,6].

A growing body of evidence shows that IPV and VAC frequently cooccur in the same families [7]. In recent years, the intersections between IPV and VAC have received increasing attention, with a growing demand for prevention efforts that target both forms of violence concurrently [8]. Intersections shared by both IPV and VAC include common risk factors, such as acceptability of family violence and parental history of physical abuse [9,10], similar short- and long-term consequences, including mental and physical health problems [11], and various intergenerational effects [12]. In the past, despite their overlaps, the fields of IPV and VAC prevention research have predominantly been separated [8]. Therefore, initiatives that seek to address these common risk factors may help shift violence prevention efforts from a siloed approach to one that targets multiple types of violence simultaneously. By targeting multiple and intergenerational forms of violence, this strategy is likely to be more sustainable long-term, helping to reduce costs and increasing opportunities for scale-up and impact [13].

In the field of IPV prevention, researchers and practitioners are increasingly adopting what is known as gender-transformative approaches in programs that aim to address multiple forms of violence, that is, both VAW and VAC [14]. More specifically, gender-transformative approaches can be defined as those that aim to address harmful social and gender norms and are “designed specifically to encourage men and boys to adopt and enact gender-equitable, nonviolent attitudes and behaviors” [15]. Many programs that adopt this approach include activities and modules that focus on topics such as improving interpersonal skills, redefining responsibilities in the home, and changing perceptions around gender roles and violence [14]. Emerging evidence suggests that gender-transformative approaches offer promising results in preventing IPV perpetration, shifting restrictive gender norms, and promoting gender-equitable behaviors and attitudes [16-18]. Gender-transformative approaches are being adopted in family and sexual health interventions, further highlighting the shift toward a more holistic approach toward violence prevention and recognition of the intergenerational elements of family violence and shared risk factors [19,20].

Alongside gender-transformative interventions, in the field of VAC, parenting programs have been identified as a major strategy for reducing and preventing violence [21]. Findings from a recent systematic review of parenting programs for reducing child maltreatment in low- and middle-income countries identified several promising results across child, parent, and family outcomes, with meta-analyses results revealing reductions in child maltreatment and harsh parenting [22]. One program that has integrated both VAC and IPV prevention content includes the gender-transformative Bandebereho program in Rwanda, which engaged men and partners with the aim of encouraging healthier couple relationships and enhancing caregiving skills [23]. The program used fatherhood as an avenue to promote gender equality and foster positive shifts in men’s relations with both their children and partners [18]. A randomized controlled trial (RCT) of the program found promising short- and long-term effects, with reductions in IPV and physical punishment of children at both 21 months posttest and at a 6-year follow-up [18].

Despite the promising potential of parenting programs to tackle multiple forms of family violence, scaling up parenting interventions remains a challenge [24]. To address barriers to scale, digital parenting interventions have been gaining momentum as a means to increase reach, engagement, and accessibility [25]. One such program, developed by Parenting for Lifelong Health [26,27], is a chatbot intervention called ParentText that provides caregivers with social learning–based parenting content that seeks to improve parent-child interaction and prevent child maltreatment [28]. Given the emerging evidence based on the potential to integrate parenting and gender-transformative approaches to prevent multiple forms of family violence, this study seeks to explore how users engage with a parenting chatbot that contains integrated IPV prevention material. Building on the development of IPV prevention material designed and integrated into the ParentText intervention in 2021 [28], the purpose of this pilot study was to examine user engagement with the IPV prevention content in the chatbot as well as explore preliminary changes in IPV.

### Objectives

The overarching aim of this pilot study is 2-fold. First, it seeks to assess parents’ and caregivers’ level of engagement with the integrated IPV prevention material in the ParentText chatbot. Second, it aims to conduct exploratory analyses of preliminary pre-post changes in experiences and perpetration of IPV and related outcomes, including attitudes toward IPV and gender-equitable behaviors following the intervention.

Consequently, this study seeks to answer the following research questions:

1. What is the level of user engagement with the IPV prevention content integrated into the parenting chatbot, ParentText, among parents and caregivers with low income in South Africa and Jamaica?
2. What, if any, are the preliminary changes in experiences and perpetration of IPV (primary outcomes) and related risk factors, including attitudes toward IPV and gender-equitable behaviors (secondary outcomes) among caregivers with low income in South Africa and Jamaica, after using the ParentText chatbot?

## Methods

### Study Setting

The study was conducted in 2 countries where IPV is particularly widespread: South Africa and Jamaica. Despite differing in their cultural contexts and geographical regions, South Africa and Jamaica share similar challenges surrounding high rates of IPV, making them comparable settings for exploring the integration of IPV prevention strategies in a parenting chatbot. South Africa has one of the highest rates of IPV globally, with studies revealing prevalence rates of 20% to 50% in which women reported having experienced IPV sometime during their life [29-31]. In Jamaica, rates of VAW are also alarming, with 24% of women having experienced physical violence, sexual violence, or both, from an intimate partner sometime in their life [32]. These high rates of violence underscore that urgent prevention efforts that seek to prevent and reduce violence are crucial. Moreover, research findings also indicate a high prevalence of patriarchal social norms and restrictive beliefs around women’s roles in both Jamaica [33] and South Africa [34], further highlighting the harmful sociocultural norms prevalent in both contexts and the overlapping challenges that both countries face. In Jamaica, this study was conducted in the parishes: Kingston, St Catherine, and St Elizabeth; and in South Africa, this study was conducted in Cape Town.

### Participants and Recruitment

In Cape Town, South Africa, participants were recruited through convenience sampling by local research assistants through the organization Clowns Without Borders South Africa. Recruitment mainly took place in neighborhoods, townships, and communities in urban Cape Town. In Jamaica, recruitment also took place through convenience sampling by members of UNICEF (United Nations Children's Fund) Jamaica staff members in partnership with the National Parenting Support Commission, an agency of the Jamaican Ministry of Education and Youth. Participants were recruited primarily at schools and at women’s centers for adolescent mothers in urban and rural communities. While the aim was to recruit both men and women, due to resource constraints, in the end, only women were recruited in Jamaica. Furthermore, while originally, the study sought to only include parents aged >18 years, following an interest expressed by UNICEF Jamaica and the Jamaican National Parenting Support Commission to address challenges faced by adolescent parents in the country, additional procedures were put in place (refer to the Ethical Considerations section for more details) to include adolescent parents aged >16 years in Jamaica. Unfortunately, due to staff and resource limitations in Cape Town, it was only possible to recruit parents aged >18 years in South Africa.

**Figure 1:**
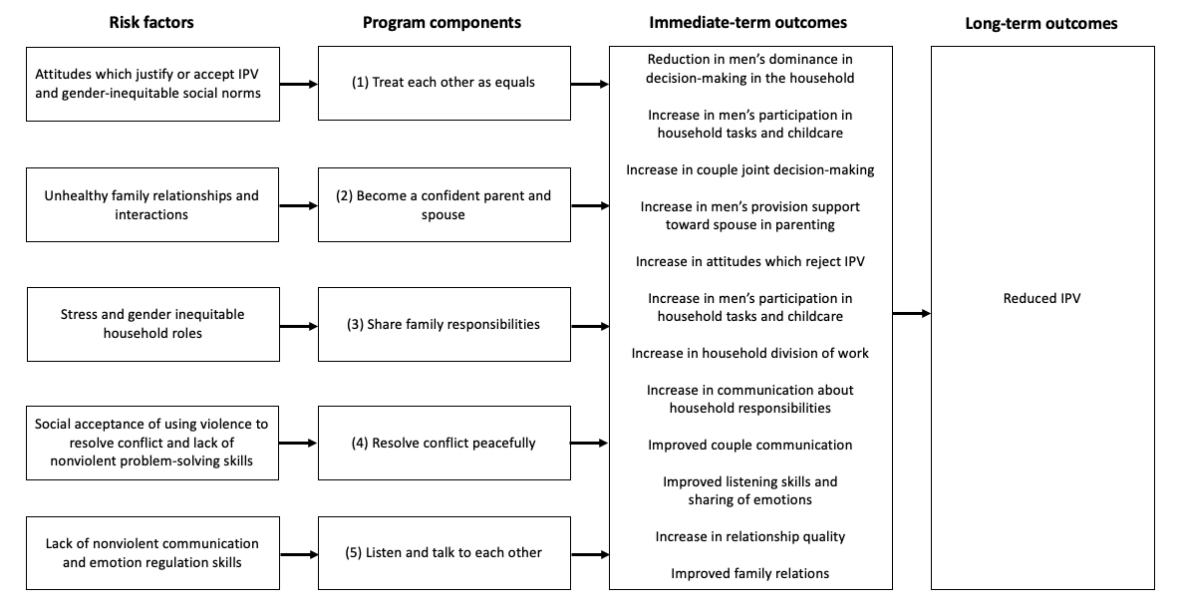
Theory of change for the ParentText intimate partner violence (IPV) prevention content, including risk factors, program components, immediate-term outcomes, and long-term outcomes.

Consequently, the inclusion criteria for participants in this study were as follows: (1) age >18 years in South Africa and >16 years in Jamaica (where adolescent parents aged >16 years were also recruited), (2) currently caring for a child aged between 0 and 17 years, (3) being in a relationship (defined as either having a partner or being married—refer to Multimedia Appendix 1 for further details), (4) having access to a phone that could receive messages on WhatsApp (Meta Platforms) or Telegram (Telegram Messenger Inc) and that could connect to 3G or the internet, (5) provided consent to participate in the study, and (6) being able to speak either English or isiXhosa (South Africa only). Exclusion criteria were participants who were not currently in a partnered relationship and participants who were not caregivers, parents, or currently caring for a child. As an initial pre-post evaluation, this study was not designed to detect significant intervention effects but rather to assess user engagement and conduct exploratory analyses of intervention outcomes [35]. Due to funding limitations, the sample size of the study was restricted to 86 participants, with some lost due to drop out (Figure 1). Nevertheless, the study used a G*Power 3 calculator with a sensitivity power analysis to calculate the effect size (Cohen *d*) needed to obtain a significant intervention effect. Input parameters included the use of 2-tailed paired *t* tests based on the study’s primary outcomes. Assuming a type I error of *P*<.05, 80% power, this sample size was sufficiently powered to detect a moderately significant intervention effect of Cohen *d*=0.67.

### Intervention

**Figure 2:**
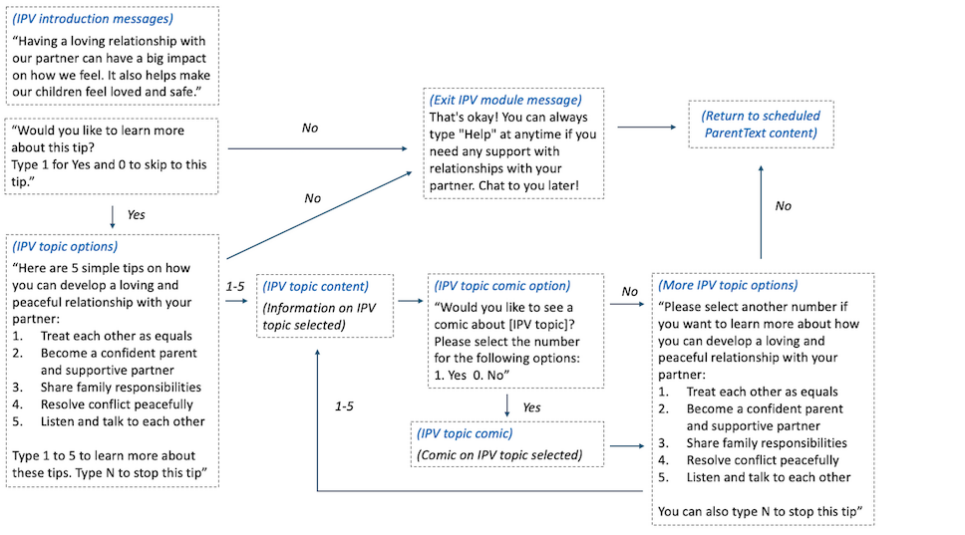
Flow of the intimate partner violence (IPV) prevention content text messages in ParentText, which illustrates the flow of text messages that users in ParentText receive, including introduction text messages and text messages providing options to view further IPV prevention content.

**Figure 3:**
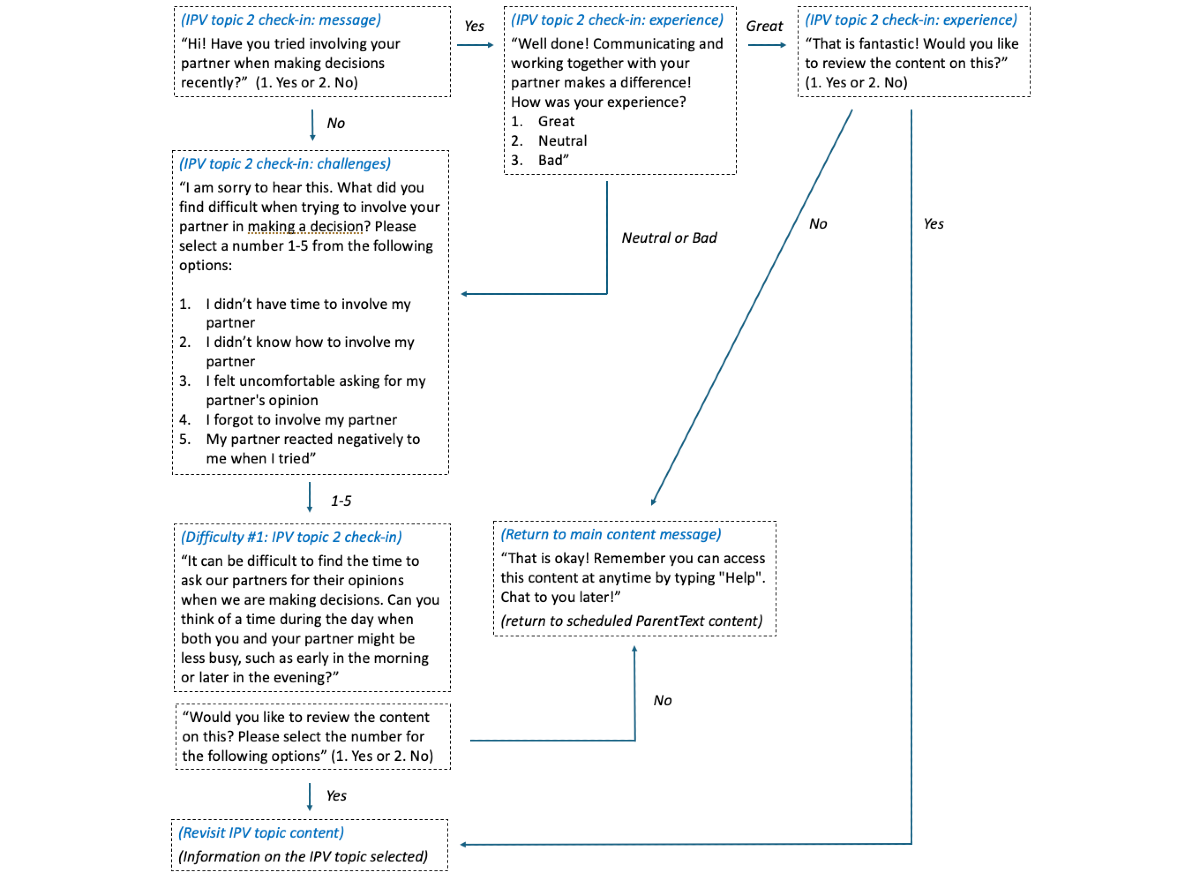
Flow of the check-in message on the intimate partner violence (IPV) topic 2 “joint decision-making,” which illustrates the flow of the check-in text message that users in ParentText receive to provide an opportunity to reflect on the topic of “joint decision-making.”.

ParentText is a chatbot intervention for parents and caregivers of children aged between 0 and 17 years [28]. The technical architecture is open source and available for developers through GitHub. The digital parenting intervention sends automated messages to users through social messaging platforms, such as WhatsApp, Telegram, and Facebook messenger (Meta Platforms), and is also available via SMS text messaging for individuals without smartphone access (for an example of program components and example messages, refer to Figures 1-3 and Multimedia Appendix 2). The parenting content of ParentText is derived from the in-person Parenting for Lifelong Health programs [26,27]. Enrolled users receive 23 days of daily ParentText messages personalized for their chosen child’s developmental stage (0-23 months, 2-9 years, and 10-17 years). Content is provided using a range of formats, including text, image, video, and audio. Structured and sequential parenting content is derived from common elements of social learning theory-based programs along two main themes as follows: (1) positive relationship building and (2) limit setting and nonviolent discipline [36]. In addition, content specifically targeting positive partner relationships and IPV prevention for users in partnered relationships was integrated into the chatbot by drawing on gender-transformative material from a range of interventions found to reduce IPV and improve gender-equitable attitudes [23,37,38]. It is the engagement with this content that is the primary focus of this study. The IPV prevention content in ParentText is divided into 5 topics (refer to program components in Figure 1). The proposed causal pathway between each IPV prevention topic, identified risk factor, and behavior change domain is illustrated in the theory of change in Figure 1. Schafer et al [28] and Multimedia Appendix 3 give further details on the development of the IPV prevention content and theory of change. Additional information and examples on how parents can interact with the chatbot, along with examples of the sequencing of the ParentText messages, are available in Figures 2 and 3.

### Design and Procedures

**Figure 4:**
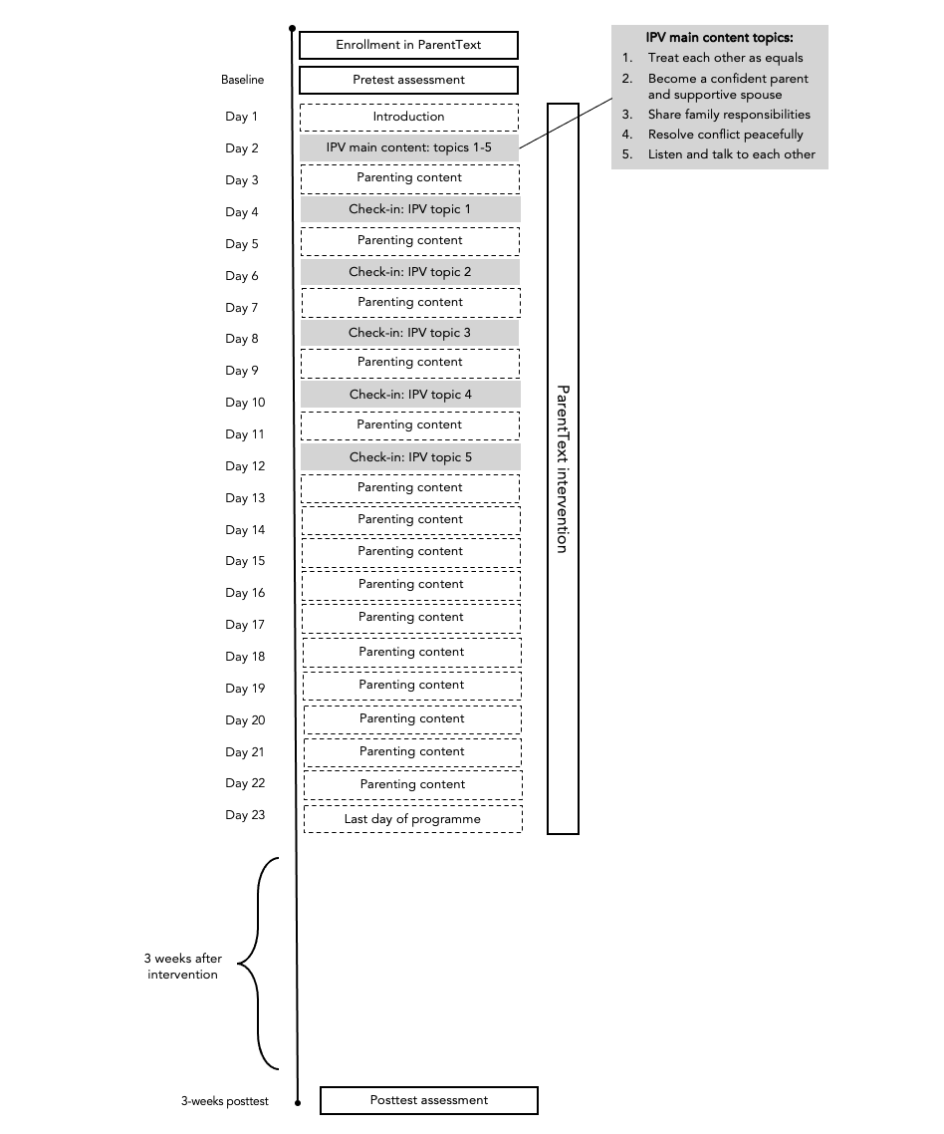
Overview of the ParentText chatbot timeline, illustrating what content is delivered on what days of the program from day 1 to day 23, and demonstrating when the 3-week posttest assessment is delivered. IPV: intimate partner violence.

This study used a repeated-measures, single-arm design. Pre and posttest assessments were conducted at baseline (preintervention) and at 6 weeks after baseline (ie, 3 weeks after the end of the intervention) using the application Open Data Kit (ODK; refer to the Measures section and Multimedia Appendix 1 for more details). The posttest was planned as a 1-month posttest (1 month after the end of intervention); however, due to fieldwork challenges that arose and time constraints, it needed to be changed to 6 weeks after baseline (Figure 4).

Reporting follows the CONSORT (Consolidated Standards of Reporting Trials) 2010 guidelines extension to randomized pilot and feasibility trials [39] (refer to Multimedia Appendix 4 for the CONSORT checklist). Recruitment, delivery, and assessments in South Africa and Jamaica were conducted on a rolling basis from September 2022 to March 2023. Participants who enrolled in the intervention received data credit using a local service provider to ensure they would have access to 3G during the intervention. During recruitment, participants received a message that included a link to the program embedded within a WhatsApp or Telegram business account. After clicking the link, participants were directed to the ParentText chatbot account and asked to select their preferred language for the program. In keeping with recommendations from the formative research [28], the language was set to English in Jamaica, and the options were English and isiXhosa in South Africa. Participants were asked if they consented to participate in the intervention and evaluation and were provided with a link to a more detailed information sheet. Participants who did not consent exited the intervention. In alignment with guidelines for conducting statistical surveys on VAW [40], the survey included skip patterns, which determine the eligibility of specific questions that respondents are asked [40,41]. For example, in this study, men who responded that their partner was taking part in the intervention were not asked the IPV items in the IPV assessment and were instead only asked the questions on attitudes toward IPV and gender roles and on gender-equitable behaviors. Further information about this is provided in the Ethical Considerations section.

At baseline, participants were invited to complete the IPV assessment, which was optional, on an external server via Oxford’s Linux virtual machine using ODK to ensure responses were not saved on their mobile devices. Participants provided their phone number when completing the IPV survey for their responses to be recorded and linked with a unique user ID. These data were then deidentified, exported as CSV files, and uploaded to a secure server in Oxford, which was only accessible by the research team and password protected. All personal identifying data were deleted once endline data collection was completed. Participants were invited to respond to the IPV assessment at 3 weeks postintervention via the phone number they provided when completing the IPV assessment at baseline.

### Measures

The user engagement, demographic, primary outcomes, and secondary outcomes are provided in Table 1.

**Table 1:** Outcome measurements in the study, including user engagement, demographics, background variables, and primary and secondary outcomes, with descriptions of the study measurement used and the number of items used for each outcome.

Outcomes		Study measurement	Items, n
**User engagement outcomes**			
	Number of days in the ParentText program	Measured by the final day the participant used the chatbot subtracted by the first day participant used the chatbot (including inactive days)	1
	Number of active days the user actively interacted with the chatbot	Number of days participants actively interacted with ParentText as measured by chatbot interactions (excluding inactive days)	1
	Overall chatbot interaction rate	The number of interactions logged per day divided by the total number of days participants actively interacted with ParentText	1
	Participant engagement with each IPV^a^ topic	The number of participants who viewed each IPV topic, in line with existing digital parenting intervention research where module viewing is frequently used as a proxy measure for the level of participant engagement [42,43]	5
	Completion rate of IPV topics	Number of IPV topics completed out of the total 5 topics (measured by the number of IPV topics participants viewed that were recorded via RapidPro, UNICEF’s open source framework developed to send and receive data using mobile phones [44])	5
	Overall completion rate of ParentText parenting modules	Number of parenting modules completed (measured by the number of modules viewed that were recorded via the RapidPro (UNICEF) software)	1
**Primary behavioral outcomes**			
	Women’s experiences of IPV	Items adapted from the WHO^b^ VAWI^c^ and the core questionnaire in the WHO-MCS^d^ [45]: physical (1 item), psychological (1 item), sexual (1 item), coercion (1 item), and economic abuse (1 item). Participants asked to give a frequency score on a scale of 0 to 8 or more times. The original VAWI tool measures past-year experiences of IPV [45]; however, a 1-year reporting time frame surpassed the duration of the intervention. The reporting timeframe and response codes were therefore adapted from the past-year (once, a few times, and many times) to the number of experiences of IPV in the past month (0, 1, 2, 3, 4, 5, 6, 7, and >8) to enhance the sensitivity of measuring changes in experiences of IPV. These adjustments are similar to those made to measure child maltreatment with the ISPCAN^e^ Child Abuse Screening Tool (ICAST)-Trial measurement by Meinck et al [46]. Past-month time frame when measuring IPV has been used in other similar pieces of research, such as a study by Ebert and Steinert [47], which examined experiences of IPV during COVID-19 in Germany. Responses were dichotomously recoded, where 1 indicated any experience of each subsequent type of IPV and 0 indicated no experience of each type of IPV in the past month [48].	5
	Men’s perpetration of IPV	Items adapted from the WHO VAWI and the core questionnaire in the WHO-MCS [45]: physical (1 item), psychological (1 item), sexual (1 item), coercion (1 item), and economic abuse (1 item). Participants were asked to give a frequency score on a scale of 0 to >8 times, with responses dichotomized into any perpetration of IPV in the past month.	5
**Secondary behavioral outcomes**			
	Men’s experiences of IPV	Two items used and adapted from Abramsky et al [49], namely: “In the past month, during any potential times that you may have used violence against your partner, did they ever fight back physically to defend themselves?” and “In the past month, have you ever been hit or physically mistreated by your partner when you were not hitting or physically mistreating them?” Respondents were asked to give a frequency score on a scale of 0 to 8 or more times, with responses dichotomized.	2
	Women’s perpetration of IPV	Two items were adapted from a questionnaire developed by researchers at the London School of Hygiene and Tropical Medicine for a randomized controlled trial of a violence prevention intervention in Tanzania [49]. Items included: “During any potential times that you were hit in the past month, did you ever fight back physically to defend yourself?” and “In the past month, have you ever hit or physically mistreated your partner when they were not hitting or physically mistreating you?” Respondents were asked to give a frequency score on a scale of 0 to 8 or more times, with responses dichotomized.	2
	Women’s positive partner interactions	Item adapted from Abramsky et al [49] asking: “How many times in the past month did your partner show you they cared and respected your feelings even though they disagreed with you?” Participants were asked to give a frequency score on a scale of 0 to 8 or more times.	1
	Men’s positive partner interactions	One item adapted from Abramsky et al [49], that asks, “How many times in the past month did you show your partner you cared and respected their feelings even though you disagreed?.” Participants asked to give a frequency score on a scale of 0 to 8 or more times.	1
	Gender-equitable behaviors	Measured using items adapted from questionnaires used in previous violence prevention interventions [23,49]. In total, 3 items were adapted from Abramsky et al [49], which included questions on couple communication, joint decision-making, and partner conflict resolution, with questions such as: “In the past week, how many times did you and your partner talk about your worries and feelings?” and “In the past week, how many times did you and your partner make a decision together?” The fourth item was adapted from a questionnaire developed by researchers of a randomized controlled trial of a gender-transformative violence prevention intervention in Rwanda [23], which asks: “In the past week, how many times did you and your partner share housework and caregiving tasks equally?” Respondents were asked to report whether in the past week the specific behavior occurred on a scale of 0 (never) to 3 (many times). The scores were summed for all items for the overall gender-equitable behavior score. The possible overall score ranged from 0 to 12.	4
	Attitudes toward gender roles and IPV	Measured using 6 items in the “Attitudes toward gender roles” section of the WHO-MCS [45], such as “A woman should obey her husband’s wishes even if she disagrees.” Respondents were asked to indicate whether they agree or disagree with the statements using a Likert scale ranging from 1 (strongly agree) to 5 (strongly disagree). Negative items were reverse-coded so that higher scores denoted more gender-equitable attitudes. The scores were summed for all items for the overall attitude score. The possible overall score ranged from 6 to 30.	6
**Background variables**			
	Parent gender, age, relationship status, living with partner, education level, employment status, partner employment status, and number of children	Sociodemographic information	8 (1 item each)
	Women’s past-year IPV experience (measured at baseline)	Measured using 1 item adapted from the WHO VAWI and the core questionnaire in the WHO-MCS [45], which asks, “How many times, in the past 12 months, did your partner do one of the following: insult or shout at you, hit or shove you, or force you to have sex?” Respondents were asked to give a frequency score on a scale of 0 to 8 or more times. Due to the skewed response patterns in the data for IPV, responses for IPV were dichotomously recoded, where 1 indicated any experience of IPV and 0 indicated no experience of IPV in the past year [48].	1

^a^IPV: intimate partner violence.

^b^WHO: World Health Organization.

^c^VAWI: Violence Against Women Instrument.

^d^MCS: Multi-Country Study on Domestic Violence.

^e^ISPCAN: International Society for Prevention of Child Abuse and Neglect.

### Statistical Analysis

Data analyses were conducted using R (R Core Team). Descriptive statistics and frequencies were used to describe the sociodemographic characteristics of participants and to report engagement outcomes, summarized using means (SDs), medians (range), and N (%). To investigate changes in outcomes between pre and posttest, McNemar chi-square tests (for dichotomous variables) and paired dependent-sample *t* tests (for continuous variables) were conducted, with corresponding 95% CIs and *P* values [50,51]. Assumptions were examined to confirm no violations existed. Data were analyzed using complete case analysis.

### Ethical Considerations

Ethical procedures were followed to ensure the safety and welfare of study participants. At the start of the IPV assessment, screening procedures ensured that men and women from the same household were not interviewed about IPV. This is based on the World Health Organization VAW research guidelines [52] and ethical and safety recommendations for intervention research on VAW [45] to prevent putting women at risk. Consequently, men who responded that their partner was taking part in the intervention were not asked the IPV items in the IPV assessment and were only asked instead the questions on attitudes toward IPV and gender roles and on gender-equitable behaviors. In addition, efforts were also in place to ensure responses to the IPV questions could not be viewed by others by delivering the IPV assessment using ODK. This procedure was implemented to protect participants by ensuring no responses were saved on their mobile devices. Participants who disclosed experiences of IPV were automatically provided referrals to local services, customized to their regional context that supported individuals experiencing violence. Local referral information could also be accessed by writing “Help Me” and choosing “Other Support” in the ParentText free text field. In line with UNICEF risk communication and community engagement guidelines [53]**,** ParentText is also formatted to detect high-risk keywords to identify potential disclosure of dangerous situations via the free text field. Following detection, ParentText automatically offers the participant an empathetic and empowering reply with referral details that are customized to the country, which supports parent and child safety (eg, hotlines, police, and ambulance).

Further ethical considerations that were taken within the research project include recognitions surrounding power and limitations of positionality [54]. For instance, it is worth noting that while collaborators and members of the research team came from several countries, the primary author of this paper is from and is part of a research institution in the global north. Efforts to address limitations associated with this positionality were put in place, including integrating reflexivity across the stages of the research process [55]. For example, during the research design and data collection planning phase, members of local grass-roots organizations, communities, and stakeholders in Jamaica and South Africa were continuously consulted to review and revise the IPV prevention content and assessment materials [28]. Integrating these embedded consultations and opportunities for research amendments with members of the local community was a pivotal part of the research project, particularly given the first author’s positionality as an “outsider” [55].

Ethics approval was obtained by the Department Research Ethics Committee at the University of Oxford (Central University Research Ethics Committee 2 Ref No: R69569/RE009). All methods were carried out in accordance with relevant guidelines and regulations. Informed consent to participate in the study was obtained from all participants before enrolling in the study and the intervention.

## Results

### Participants Characteristics

**Figure 5:**
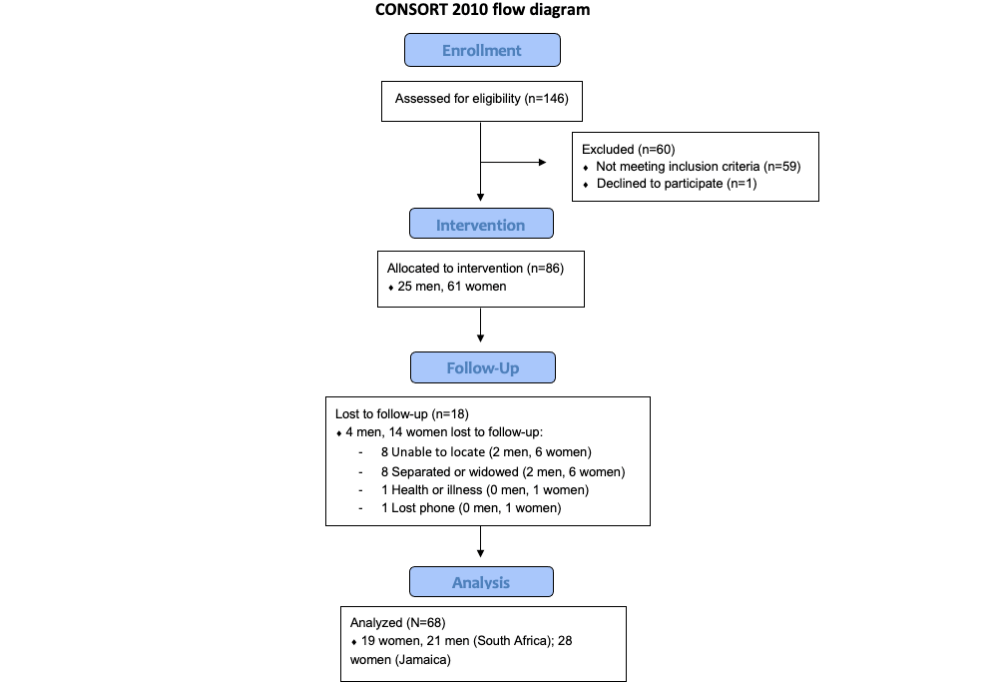
Study flow diagram illustrating the flow of participants through the trial. CONSORT: Consolidated Standards of Reporting Trials.

Participant characteristics are provided in Table 2 by country and with an overall summary. A study flow diagram illustrating the flow of participants through the trial is provided in Figure 5. The average age of participants across both countries was 34.4 (SD 8.74; range 16-57) years. The average age of women in South Africa was 34.9 (SD 6.77; range 25-49) years and men 28.8 (SD 2.68; range 25-36) years. In Jamaica, where only women were recruited into the study, the average age of women was 39.9 (SD 11.52; range 16-57) years. Most participants, across both countries, were partnered but not married (48/68, 71%). On average, participants had 2.23 (SD 1.19) children. Approximately half of the participants across both countries had a secondary education (36/68, 53%), and 7% (5/68) had only primary education or less. In South Africa, among women participants, 42% (8/19) were working and 58% (11/19) were unemployed or looking for work. Among men, slightly more were working (13/21, 62%), with 29% (6/21) unemployed or looking for work. In Jamaica, among women participants, this was similar, with 43% (12/28) working and 54% (15/28) unemployed or looking for work. Across both countries, the employment of partners was high (50/68, 74%), with only 18% (12/68) with partners who were unemployed or looking for work. Experience of IPV (women-report only due to safety reasons) at baseline was high: 21% (4/19) of women participants in South Africa and 29% (8/28) of women in Jamaica had experienced some form of IPV in the past year, respectively.

**Table 2:** Demographic characteristics of study participants in South Africa and Jamaica.

		South Africa		Jamaica	Overall
		Women (n=19)	Men (n=21)	Women (n=28)	Total (N=68)
**Gender, n (%)**					
	Women	19 (48)	N/A^a^	28 (100)	47 (69)
	Men	N/A	21 (53)	—^b^	21 (31)
Age (y), mean (SD)		34.9 (6.77)	28.8 (2.68)	39.9 (11.52)	34.4 (8.74)
**Relationship, n (%)**					
	Married	6 (32)	2 (10)	12 (43)	20 (29)
	Partnered but not married	13 (68)	19 (90)	16 (57)	48 (71)
Number of children, mean (SD)		2.53 (1.17)	1.19 (0.68)	2.61 (1.25)	2.23 (1.19)
**Education, n (%)**					
	Primary	1 (5)	0 (0)	4 (14)	5 (7)
	Secondary	9 (47)	10 (48)	17 (61)	36 (53)
	Higher	9 (47)	11 (52)	7 (25)	27 (40)
**Employment, n (%)**					
	Working	8 (42)	13 (62)	12 (43)	33 (49)
	Unemployed or looking for work	11 (58)	6 (29)	15 (54)	32 (47)
	Student	0 (0)	2 (10)	0 (0)	2 (3)
	Retired	0 (0)	0 (0)	1 (4)	1 (2)
**Partner’s employment, n (%)**					
	Working	11 (58)	17 (81)	22 (79)	50 (74)
	Unemployed or looking for work	7 (37)	2 (10)	3 (11)	12 (18)
	Student	0 (0)	2 (10)	0 (0)	2 (3)
	Retired	0 (0)	0 (0)	2 (7)	2 (3)
	Refuse to answer	1 (5)	0 (0)	1 (4)	2 (3)
**Experience of IPV^c^, n (%)**					
	Experienced IPV in the past year^d^	4 (21)	X^e^	8 (29)	12 (26)^f^

^a^N/A: not applicable.

^b^No men were recruited in Jamaica.

^c^IPV: intimate partner violence.

^d^For safety reasons, men were not asked this question at baseline.

^e^Men were not asked this question at baseline due to safety reasons.

^f^Overall IPV here only includes women.

### User Engagement Outcomes

Overall engagement outcomes are provided in Table 3. The ParentText program lasted 23 days, with the main IPV prevention content delivered on day 2 and check-in messages delivered within the first 12 days (Multimedia Appendix 2 provides an overview of the program content delivered each day). In terms of engagement, in South Africa, the average number of days in the program was 13 (SD 23.90) and 3 (SD 3.78) days for women and men, respectively. The average number of days users interacted with the chatbot was 3 (SD 1.58) for women and 2.6 (SD 1.52) for men, each with an average interaction rate of 0.57 (SD 0.26) interactions and 0.59 (SD 0.17) interactions per day, respectively. In Jamaica, the average number of days in the program was 27 (SD 23.10) days, and the average total number of days users interacted with the chatbot was 5.2 (SD 5.45) days. The average interaction rate in Jamaica was 0.21 (SD 0.20) interactions per day. In terms of engagement across the 5 relationship topics, there was a greater engagement with topics in Jamaica where engagement with each topic ranged from 11% (3/28) to 29% (8/28) compared to South Africa, where it ranged from 0% to 11% (2/19). In terms of completion rates, in South Africa, 25% (5/20) and 5% (1/20) of women and men completed 1 out of the 5 IPV topics, and 5% (1/20) and 0% completed all 5 IPV topics, respectively. In comparison to the completion rate of overall ParentText modules, 16% (3/19) and 9% (2/21) of women and men completed all the parenting (ie, the non-IPV) ParentText modules in South Africa. In contrast, in Jamaica, 21% (6/28) completed 1 out of the 5 IPV topics, 11% (3/28) completed all 5 IPV topics, and 18% (5/28) completed all the parenting ParentText modules.

**Table 3:** ParentText user engagement outcomes of the 23-day program in South Africa and Jamaica, including overall retention, engagement per intimate partner violence (IPV) topic, and IPV topic completion rate.

Outcomes				South Africa				Jamaica
				Women (N=19)		Men (N=21)		Women (N=28)
**Overall retention, mean (SD)**								
	Number of days in program^a^			13 (23.90)		3 (3.78)		27 (23.10)
	Number of active days the user interacted with the chatbot^b^			3 (1.58)		2.6 (1.52)		5.2 (5.45)
	Number of interactions per day^c^			0.57 (0.26)		0.59 (0.17)		0.21 (0.20)
**Engagement per IPV topic^d^**								
	**Participants who viewed each IPV topic,** **n (%)**							
		Relationship topic 1: “Treat each other as equals”	2 (11)		2 (10)		7 (25)	
		Relationship topic 2: “Become a confident parent and supportive spouse”	2 (11)		0 (0)		4 (14)	
		Relationship topic 3: “Share family responsibilities”	2 (11)		1 (5)		5 (18)	
		Relationship topic 4: “Resolve conflict peacefully”	2 (11)		1 (5)		3 (11)	
		Relationship topic 5: “Listen and talk to each other”	2 (11)		1 (5)		8 (29)	
		All relationship topics (viewed all relationship topics)	1 (5)		0 (0)		3 (11)	
	**Completion rate of IPV topics completed, n (%)^e^**							
		1	5 (25)		1 (5)		6 (21)	
		2	0 (0)		0 (0)		3 (11)	
		3	0 (0)		0 (0)		1 (4)	
		4	0 (0)		1 (5)		0 (0)	
		5	1 (5)		0 (0)		3 (11)	
	Completion rate of ParentText parenting modules, mean (SD)			16 (0.19)		9 (0.06)		18 (0.20)

^a^Measured by the final day participant used the chatbot, subtracted by the first day the participant used the chatbot (note: this measure includes inactive days).

^b^Number of days participants actively interacted with ParentText (note: this measure excludes inactive days).

^c^Measured by the number of interactions logged per day divided by the total number of days the participant actively interacted with ParentText.

^d^Measured based on the number of participants who viewed each IPV topic.

^e^Number of IPV topics completed out of the total 5 topics.

### Behavioral Outcomes

#### Experiences and Perpetration of IPV

Table 4 presents the descriptive results of the IPV outcomes, summarizing experiences of IPV among women and perpetration of IPV among men using past-month frequencies at pre and posttest and *P* values for each outcome. At posttest, 73% (11/15) of women in South Africa and 72% (13/18) of women in Jamaica reported experiencing some form of IPV, compared to 84% (16/19) and 96% (23/24) at baseline, reductions that were significant in South Africa (*P=*.01) and in Jamaica (*P*=.01). Experiences of all subtypes of IPV for women in both South Africa and Jamaica also showed reductions at posttest, and so did acts of self-defense and perpetration (Multimedia Appendix 5). For perpetration of IPV among men, at posttest, 75% (9/12) of men in South Africa perpetrated some form of IPV, compared to 85% (11/13) at baseline, which was nonsignificant for overall perpetration of IPV (*P*=.06). Analyses of subtypes of IPV, however, found significant reductions in psychological (*P*=.04), physical (*P*<.001), and sexual (*P*<.001) violence among men in South Africa (Multimedia Appendix 5). Detailed tables of the primary and secondary outcomes are provided in Multimedia Appendix 5.

**Table 4:** Primary behavioral outcomes for women’s past-month experience of overall intimate partner violence (IPV) in South Africa and Jamaica and men’s past-month perpetration of overall IPV in South Africa.

		Baseline overall IPV, n (%)^a^	Posttest overall IPV, n (%)	*P* value^b^
**Women’s experience of overall IPV (past month)**				
	South Africa	16/19 (84)	11/15 (73)	.01
	Jamaica	23/24 (96)	13/18 (72)	.01
**Men’s perpetration of overall IPV (past month)**				
	South Africa	11/13 (85)	9/12 (75)	.06

^a^% used is proportional to the baseline and posttest samples, respectively.

^b^McNemar chi-squared *P* value.

#### Positive Partner Interactions

Table 5 presents outcomes on positive partner interactions measured through respectful behaviors, summarized using means and SDs for pre and posttest along with *P* values. There were no significant effects detected for change in positive partner interactions reported by men or women.

**Table 5:** Secondary behavioral outcomes for positive partner interactions in the past month, overall attitude toward gender roles and intimate partner violence (IPV), and overall gender-equitable behaviors in the past week for women and men in South Africa and women in Jamaica.

				Baseline, mean (SD)		Posttest, mean (SD)		*P* value (*t* test)
**Positive partner interactions**								
	**South Africa**							
		Women (n=15)	4.74 (2.84)		4.14 (2.98)		.57	
		Men (n=12)	3.92 (2.50)		5.67 (2.46)		.10	
	**Jamaica**							
		Women (n=15)	5.08 (2.60)		3.89 (2.54)		.14	
**Overall attitude^a^ toward gender roles and IPV**								
	**South Africa**							
		Women (n=14)	10.11 (2.71)		10.13 (3.89)		.98	
		Men (n=16)	11.90 (3.82)		8.88 (2.63)		.01	
	**Jamaica**							
		Women (n=15)	11.39 (3.45)		11.89 (2.35)		.56	
**Overall gender-equitable behaviors^b^**								
	**South Africa**							
		Women (n=15)	7.37 (2.95)		7.93 (3.53)		.63	
		Men (n=16)	7.81 (1.94)		9.75 (1.61)		.02	
	**Jamaica**							
		Women (n=15)	7.14 (3.49)		7.39 (2.85)		.80	

^a^A lower score indicates less harmful attitudes toward IPV and gender roles.

^b^A higher score indicates more gender-equitable behaviors in the past week.

#### Attitudes Toward (Harmful) Gender Roles and IPV

Table 5 summarizes means and SDs for the attitude outcomes at pre and posttest, with *P* values for each attitude outcome also provided. Analyses indicate a significant reduction in harmful attitudes toward IPV and gender roles reported by men in South Africa (*P*=.01); however, no effects were reported among women in South Africa (*P*=.98) or in Jamaica (*P*=.56).

#### Gender-Equitable Behaviors

Analyses found a significant increase in gender-equitable behaviors for men (*P*=.02; Table 5). No significant effects were detected for women in South Africa (*P*=.63) or in Jamaica (*P*=.80).

## Discussion

### Principal Findings

To the best of our knowledge, this pre-post pilot study is the first that examines user engagement and indicative outcomes in a digital parenting intervention with integrated IPV prevention content [22]. The study offers valuable, preliminary insights across 2 countries on user engagement with a digital parenting intervention with integrated IPV prevention, as well as provides exploratory, indicative results on IPV experience and perpetration, attitudes toward IPV, and gender-equitable behavior outcomes. First, we will discuss the key findings on levels of user engagement. While the average daily interaction rate was higher in South Africa (0.57 and 0.59 interactions per day for women and men, respectively) compared to Jamaica (0.21 interactions per day), the average number of days users stayed in the program in Jamaica was greater (27 days), compared to South Africa (13 and 3 days for women and men, respectively). Due to WhatsApp messaging restrictions, “push” messaging was paused after 24 hours of user inactivity, until user activity resumed. Given research findings that highlight the importance of messaging reminders in digital interventions [56], this restriction may have contributed to some of the low engagement levels observed.

The completion rate of engaging with at least 1 IPV topic in the program was lower than expected across the intervention, with completion rates of 5% (1/20) for men and 25% (5/20) for women in South Africa, and 21% (6/28) for women in Jamaica. The completion rates of all 5 IPV topics were much lower, with 0% of men and 5% (1/20) of women in South Africa, and 11% (3/28) of women in Jamaica completing all 5 IPV prevention topics. Even though user engagement and retention rates have been noted as a challenge in digital interventions [57], completion rates in this study were lower than anticipated in comparison to findings in the field. For example, a review of digital interventions for parents with young children found that attrition rates in digital parenting programs ranged from 30% to 50% [58]. Accordingly, the low completion rates of the IPV topics observed in ParentText raise questions about whether user engagement in the program was sufficient to change complex behaviors and deeply rooted attitudes surrounding violence and gender roles.

There are various possible reasons for the low levels of engagement with the IPV content observed in ParentText. First is the format of the program schedule. On day 2 of the ParentText program, participants were provided with a list of all 5 IPV prevention topics, which they could choose to explore. After selecting a topic to view, users were given the option to view one of the other IPV topics. However, it is possible that viewing >1 IPV prevention topic per day may have been overwhelming for users. Therefore, this may have inhibited users from engaging with multiple IPV topics in the program. Another element that may have affected engagement was the timing of the program “check-in” reminders. Participants received “check-in” messages in the latter part of the program (days 4-12; Multimedia Appendix 2), where they were encouraged to revisit and explore the IPV topics they had not yet engaged with. However, given the already low average number of days for which the participants remained in the program, it is likely that many participants did not receive these messages. These findings raise important questions on how to increase engagement with the IPV modules integrated into the ParentText. For example, amending the timing of content delivery might be one way to increase engagement. For example, a study assessing a text-messaging intervention focusing on promoting health relationships found that the timing of message delivery played a key role in program engagement and that if delivered at the wrong time, users might be less likely to engage with the text messages [59].

Taken together, these study results give valuable insight into how users engage with IPV content integrated into a chatbot and suggest that rather than interacting with the chatbot content daily, users appear to interact with the chatbot at their own pace. Consequently, providing more options for user-led selection of program content would be important for future iterations of the chatbot to ensure users can access and revisit the information they need more readily. The importance of user-led content delivery has also been underscored by other digital parenting intervention research. For instance, findings from a study on the digital fatherhood program “SMS4dads” revealed that the length of time between users receiving a text message with a link to program content to that of users clicking the link was, on average, 2.11 (SD 3.94) days [60]. This highlights how engagement with digital intervention content is often not instantaneous, but rather asynchronous and driven by users’ schedules [60]. Accordingly, in future versions of ParentText, combining “push” notification reminders with user-led selection of program content might help increase user engagement. Notably, since the present trial of ParentText in South Africa and Jamaica, newer versions of the WhatsApp settings have been released, which allow future iterations of the program to override the 24-hour limit inhibiting “push” messages after user inactivity.

More broadly, the relatively low levels of user engagement found in this pilot study raise questions as to whether a digital modality is sufficient when delivering intervention content related to complex gender-equitable beliefs and behaviors. It may be the case that a hybrid approach, combining both digital content delivery and in-person sessions, might be necessary. For instance, a study on enhancing father engagement in parenting programs revealed that in terms of intervention delivery, fathers prefer less intensive and low-dose programs, including internet‐based interventions [61]. However, a recent systematic review of interventions promoting gender equality noted that in various studies it was found that dialog was critical for driving change in gender norms [62]. The importance of dialog is further underscored by research examining men’s engagement in family and health interventions, revealing that men value and often seek the opportunity to connect with other men to discuss parenting and fatherhood, even though cultural norms sometimes hinder this. Together, these findings suggest that a combination of digitally delivered content and in-person discussion opportunities might be needed. Various studies also suggest that using face-to-face recruitment and participatory, peer-engaged methods is an effective approach for engaging parents, particularly fathers [63,64]. Research findings also suggest that providing opportunities to critically discuss and engage in dialog with others, especially on difficult topics, plays a valuable role not only in terms of engagement but also in terms of creating change and shifting harmful gender attitudes [17,62,64]. Taken together, it may be possible that low-intensity, 1-way engagement with a chatbot is insufficient to create change in more deeply rooted behaviors and attitudes among users and that more interactive engagement is needed to shift behaviors and beliefs. While these hybrid options and amendments may improve engagement, it should be noted that they will also increase intervention costs and limit scalability, which are some of the benefits of digital-only interventions [24].

It is also possible that the low level of user engagement with the IPV prevention topic was due to other factors, such as the format of the program. The IPV content in ParentText was only delivered on 1 specific day, after which only check-in messages were sent (refer to Figure 4 for an overview of the content delivery schedule). Receiving the option to view all the IPV content on the same day may potentially have been overwhelming for participants. This may have reduced user engagement rates due to intervention fatigue [65], which is cognitive or emotional weariness resulting from competing demands of intervention engagement and other burdens in daily life [66] and has been linked to intervention adherence. Considerations for future iterations of ParentText include (1) delivering all the content automatically (since optional and supplemental content in digital interventions is often associated with low engagement [67]) and (2) adjusting the timing of content and spreading out the delivery of the IPV content to allow mental rests in between new material, strategies that have been suggested to reduce intervention fatigue [65]. Notably, this is already an amendment that future iterations of ParentText are incorporating and that is currently being implemented in a trial of an updated version of ParentText in Malaysia [68]. This integrated approach of implementing IPV prevention content beyond a single module is also a strategy that is favored and advocated by gender-transformative approaches and is one that is being advocated more broadly in the parenting field [69].

The exploratory analyses of the behavioral outcomes, while only preliminary, are also important to discuss. In terms of IPV, women in Jamaica reported a significant reduction in overall experiences of IPV at posttest. These tentative findings are in line with results from other parenting and gender-transformative programs that have found reductions in both IPV experience at posttest and even at follow-up [18,23,37]. For example, findings from an RCT of a gender-transformative intervention in Rwanda found that, compared to the control, women in the intervention group reported reductions in both physical and sexual IPV following the intervention [23].

In contrast to the significant reductions in IPV experience among women, no significant effects for overall IPV perpetration were detected among men in South Africa. However, analyses of IPV subtypes found significant reductions in psychological violence, physical violence, and sexual violence among men in South Africa (Multimedia Appendix 5). The lack of change in overall IPV perpetration might be related to the low level of male engagement. Notably, the user engagement and the IPV completion rate of men in South Africa were 50% less than those of women. Hence, the limited interaction men had with the chatbot may not have been sufficient to change these behaviors.

It may also be the case that more interactive engagement is needed to shift these complex behaviors. For instance, emerging findings suggest that content modality can impact digital intervention outcomes [42], as highlighted in a recent study on ChattyCuz, a chatbot intervention that aims to support young women in navigating intimate relationships in South Africa [70]. Interestingly, an RCT of ChattyCuz found that while the gamified version of the chatbot (treatment 1) led to modest but significant reductions in IPV experience compared to those without treatment, in the narrative treatment, there was no effect on IPV experience [70]. The authors hypothesize that the lack of measurable effect on IPV experience in the narrative treatment arm of the study might be related to the lack of user interaction, that greater engagement with intervention content is necessary for behavior change, and that an information-only approach is insufficient [70]. These findings highlight how low levels of user engagement in ParentText might play a role in the lack of behavior changes detected and underscore that, amendments to the program modality might be needed to make the content more interactive to shift behaviors. Recent studies have also demonstrated the strong impact that interpersonal relationships have on gender attitudes, underscoring the need for interventions to not only target individuals but also social networks, such as peers and family members through, for example, critical reflection and dialog among social groups [71]. Research suggests that programs that have shown evidence of change in gender norms involve the engagement of stakeholders at different levels of the social-ecological model, include activities that allow for active participation, and promote critical awareness [14]. Consequently, it is possible that greater interactive engagement is needed to see greater changes in IPV experience and perpetration.

This study also revealed a significant overall reduction in harmful attitudes toward IPV as well as a significant increase in overall gender-equitable behaviors among men in South Africa. These indicative effects are promising, and while caution is warranted given the small sample, the findings align with other interventions that have observed reductions in harmful attitudes among men at posttest and detected increases in gender-equitable behaviors following gender-transformative interventions [72,73].

### Strengths and Limitations

The study has various strengths worth noting. As the first study of a digital parenting intervention with integrated IPV prevention content, the present research makes a valuable contribution to the field of violence prevention and targeted efforts seeking to address VAC and IPV concurrently. With a growing emphasis in policy and research on preventing multiple forms of violence simultaneously [74], this study sheds light on preliminary behavioral outcomes and how users engage in IPV prevention content that has been integrated into a digital parenting intervention. Another strength of the study is the use of data from 2 countries. By delivering the program in 2 different regions and cultures, this study offers an insight into important considerations to bear in mind before scaling up the intervention. For instance, the challenges with recruiting men in Jamacia compared to South Africa suggest that alternative recruitment strategies might be needed when scaling up the intervention in the Caribbean region.

The study also has various limitations. The first relates to the lack of a control group, without which causality cannot be established from the pre-post data. The study also had a small sample size, which means that the research findings need to be interpreted with caution given that there is limited statistical power in detecting pre-post intervention effects. Consequently, the next step is a fully powered RCT to establish effectiveness. Future research would also benefit from incorporating a factorial experiment design, which can be used to examine which specific intervention components have an effect on outcomes and which components interact with each other [75]. Knowing which components should be kept to sustain impact when taking an intervention to scale or delivering it in a new setting is critical, especially when considering program length and implementation costs [76]. Including follow-up assessments to examine whether intervention effects remain, become reduced, or are delayed over time [77] would also be beneficial in future studies as this study was limited to a 6-week posttest due to resource and time constraints. In addition, the study used a convenience sampling approach, which may limit the generalizability of the study findings. For example, it is possible that caregivers who opted to take part in the parenting program were more willing to change, which may bias the results. The outcomes in the study were also self-reported, and consequently, they may be subject to disclosure bias, recall bias, and social-desirability bias, leading to under reporting of unfavorable behaviors and over reporting of what might be perceived as desirable answers. However, given that the self-reports were carried out on participants’ phones and participants were told the reports were fully anonymized, this will likely have reduced desirability bias in the quantitative surveys.

Another important limitation is that while in this study, participants were provided with mobile credit, this may not always be realistic in a real-world setting. While access to the internet and mobile data are increasing globally [78], it is important to explore potential options of being able to use the program without mobile data, especially when planning strategies for scale-up and delivery. One digital intervention that has explored this is the parenting program ParentApp for Teens, which is a mobile app designed for offline use that targets parents of teenagers [79,80]. There are also assessment limitations related to the self-reported measurements used in this study. Given the sensitive nature surrounding IPV and attitudes toward violence, it is possible that participants were hesitant to disclose violence due to stigma or fear of being endangered, leading to under reporting and consequently leading to undetected changes in behaviors [81]. Similarly, social-desirability bias may have also impacted the measurement of behavioral outcomes, particularly the measurement of IPV perpetration, which has been found to be more prone to social-desirability bias than reports of IPV experience [82].

An additional limitation worth noting is that men were underrepresented in the study. Recruitment of male caregivers is a known difficulty with parenting interventions [83], and even though it was possible to recruit both male and female caregivers in South Africa, it was only possible to recruit female caregivers in Jamaica. The lack of male caregivers from Jamaica means that certain experiences may have been underestimated due to the low number of fathers included. This challenge highlights the need for future studies to ensure greater efforts are taken to adopt recruitment strategies that ensure men are also included. In future studies, it would also be valuable to include people aged between 16 and 17 years in the South African sample to better understand how adolescent parents in South Africa engage with the chatbot.

### Conclusions

This research presents tentative findings on the first-ever attempt to research the integration of IPV and parenting content into a digital intervention. Some of the preliminary results of the intervention are promising, with indications of reductions in overall IPV for women and a potential decrease in harmful IPV attitudes among men. However, the high rates of IPV experiences and perpetration reported at the posttest are still concerning and suggest that greater intervention efforts and programmatic amendments are likely necessary to tackle deep-rooted attitudes and harmful behaviors. In addition, the unexpectedly low levels of engagement in the intervention raise questions about whether interactions with the chatbot were sufficient to shift behaviors and attitudes and suggest that a hybrid approach to program delivery might be necessary. Given the urgent need for scalable prevention efforts that can tackle multiple forms of violence concurrently [84], more research is necessary to further explore how to increase chatbot user engagement and achieve greater reductions in IPV and harmful attitudes toward gender roles and IPV.

## Appendix

Multimedia Appendix 1 Intimate partner violence assessment.

Multimedia Appendix 2 Structure of the ParentText intervention.

Multimedia Appendix 3 Overview of ParentText intimate partner violence prevention content.

Multimedia Appendix 4 CONSORT (Consolidated Standards of Reporting Trials) checklist.

Multimedia Appendix 5 Detailed primary and secondary outcome tables.

## Abbreviations

CONSORT: Consolidated Standards of Reporting Trials
IPV: intimate partner violence
ODK: Open Data Kit
RCT: randomized controlled trial
UNICEF: United Nations Children’s Fund
VAC: violence against children
VAW: violence against women

## Data Availability

The datasets generated and analyzed during this study are available from the corresponding author on reasonable request.
