# Supplementary material for: A Digital Parenting Intervention With Intimate Partner Violence Prevention Content: Quantitative Pre-Post Pilot Study
Source: JMIR Form Res. 2025 Jan 3;9:e58611. doi: 10.2196/58611 (PMC11748420; doi:10.2196/58611)
Supplement: Multimedia Appendix 1 [file formative_v9i1e58611_app1.docx]

#### Multimedia appendix 1. IPV assessment

At the start of the IPV Assessment, participants were asked to provide details on their family composition, age, gender, and additional sociodemographic characteristics. All personal identifying data were deleted once endline data collection was completed. The IPV assessments were optional, and participants were reminded their answers were completely private and that they could refuse to answer and still receive ParentText messages.

IPV Assessment

**[Invitation via RapidPro to participate in the IPV survey]:**

[Invitation to participate in external survey]:

Can we ask you a few more quick questions about your relationship with your partner? Everything you say will be completely private.

If you do not want to answer any questions, it is completely okay.
Type "1" if you are happy to answer some questions. Type "0" if you want to skip this.

[Response to "0"]:
That’s okay! It is alright if you do not want to answer these questions. Chat to you later!

[Response to "1"]:
To answer these questions, click this link to take you to an external site for extra privacy: [URL]

**[IPV survey via an external secure server]:**

[Assessment Introduction]

**Our Relationships**

**Introduction [Women and Men]**

Thank you very much for taking the time to complete this survey about relationships!

Remember that your name, telephone number, and answers to these questions will be kept private. If there are any questions you do not feel comfortable answering, you do not have to answer them.

- Please enter today's date

**About You [Women and Men]**

- What is your telephone number?
  If you have multiple phone numbers, please use the one linked to the parenting text messages (e.g. WhatsApp number if receiving messages via WhatsApp)
- What is your gender?

1. Female
2. Male
3. Other

- What is your relationship status?^[[1]](#footnote-1)^

1. Single (not in a relationship)
2. Married
3. Partnered but not married^[[2]](#footnote-2)^
4. Divorced or separated
5. Widowed

- Are you currently living with your partner?

(Choice options: Yes; No; Don’t know)

- How old are you in years?
  (Choice option: *Open numeric answer*)
- What is the highest level or grade you have completed at school?

1. Never went to school
2. Primary
3. Secondary
4. Higher
5. Don’t know
6. Refuse to answer

- What is your current employment status?

1. Working
2. Unemployed / looking for work
3. Retired
4. Student
5. Refuse to answer

- What is your partner's current employment status?

1. Working
2. Unemployed / looking for work
3. Retired
4. Student
5. Refuse to answer

- How many children do you have or care for (including those who may not be directly yours)?
  (Choice option: *Open numeric answer*)
- Is your partner also receiving ParentText messages on their own device?* [*only men are asked this question]
  (Choice options: Yes; No; Don’t know)

[Section 1A: Attitudes and Beliefs]

**Views and beliefs [Women and Men]**

In the community and beyond, people have different ideas about families and what is acceptable behaviour for men and women in the home. For the following statements I would like your views on what is acceptable. There are no right or wrong answers.

Please indicate whether you agree or disagree with the following statements:

(Strongly Agree=1; Agree=2; Neutral=3; Disagree=4; Strongly Disagree=5; Refuse to answer)

1. A woman should obey her husband’s wishes even if she disagrees.
2. A couple should decide together things that affect the health and well-being of the family.
3. Fathers would benefit if they were more involved in caring for their children.
4. It is natural and right that men have more power than women in the family.
5. A man has a good reason to hit his wife if she disobeys him.
6. It’s a wife’s obligation to have sex with her husband even if she doesn’t want to.

[Section 1B: Relationships]

**Our Relationships [Women and Men]**

When two people are in a relationship or live together, they usually have both good and bad moments. For the following questions, think about things you may have experienced with your partner. (Choice options: Never; Once; Few times; Many times; Refuse to answer)

- In the past week, how many times did you and your partner talk about your worries and feelings?
- In the past week, how many times did you and your partner make a decision together?
- In the past week, how many times did you and your partner share housework and caregiving tasks equally?
- In the past week, how many times did you explain your side of a disagreement to your partner in a respectful way?

[Section 2: IPV Survey + Referral Info] [Women]

**Experiences [Women]**

Being in a relationship with another adult can be challenging. No matter how well adults get along, there are times when they want different things and disagree. Some of the following questions might be hard. Please feel free to answer as truthfully as possible. Remember nobody in your family or community will ever see these answers. For the following questions, think about things you may have experienced from your partner IN THE PAST MONTH. (Choice options: 0, 1, 2, 3, 4, 5, 6, 7, 8+ times; Refuse to answer).

- How many times in the past month did your partner show you they cared and respected your feelings even though they disagreed with you?
- How many times in the past month did your partner insist on knowing where you were at all times?
- How many times in the past month did your partner refuse to give you money for household expenses, even when they had money for other things?
- How many times in the past month did your partner insult, shout, yell, or swear at you?
- How many times in the past month did your partner slap, shove, push, or hit you?
- How many times in the past month did your partner use threats or force to make you have sex?

We appreciate your honesty in responding to these questions. We would now like to ask about your experiences in the PAST 12 MONTHS. (Choice options: 0, 1, 2, 3, 4, 5, 6, 7, 8+ times; Refuse to answer).

- How many times, in the past 12 months, did your partner do one of the following: insult or shout at you, shove or hit you, or force you to have sex?

It is common for adults who are in a relationship to have both good and bad moments. For the following questions, think about how your partner’s actions may have impacted you. For these questions think about your experiences with your partner IN THE PAST MONTH. (Choice options: 0, 1, 2, 3, 4, 5, 6, 7, 8+ times; Refuse to answer).

- During any potential times that you were hit in the past week, did you ever fight back physically to defend yourself?
- In the past week, have you ever hit or physically mistreated your partner when they were not hitting or physically mistreating you?

**Thank you! [End of IPV Assessment – Women]**

Thank you very much for answering these questions! We really appreciate it.

You have the right to be treated with dignity and respect, and live free from fear and violence. If you or someone you know is experiencing any type of abuse, there are many organisations that can help you:

- Domestic Abuse National Hotline: [telephone]
- Link to domestic abuse support services in your area: [URL]
- If you are in an emergency situation, call [999]

[Section 2: IPV Survey + Referral Info] [Men*]

*[*men are only assessed when their partner is not using the chatbot]*

**Experiences [Men]**

Being in a relationship with another adult can be challenging. No matter how well adults get along, there are times when they disagree, get frustrated with each other, have difficult moments, or want different things. Some of the following questions might be hard. Please feel free to answer as truthfully as possible. Remember nobody in your family or community will ever see these answers. For the following questions, think about things you may have experienced from your partner IN THE PAST MONTH. (Choice options: 0, 1, 2, 3, 4, 5, 6, 7, 8+ times; Refuse to answer)

- How many times in the past month did you show your partner you cared and respected their feelings even though you disagreed?
- How many times in the past week did you insist on knowing where your partner was at all times?
- How many times in the past month did you refuse to give your partner money for household expenses, even when you had money for other things?
- How many times in the past month did you insult, shout, yell, or swear at your partner?
- How many times in the past month did you slap, shove, push, or hit your partner?
- How many times in the past month did you use threats or force to make your partner have sex?

Remember that your answers will be completely private. Nobody in your family or home will see them. We would now like to ask about your experience in the PAST 12 MONTHS. (Choice options: 0, 1, 2, 3, 4, 5, 6, 7, 8+ times; Refuse to answer).

- How many times, in the past 12 months, did you: insult or shout at your partner, hit or shove your partner, or force your partner to have sex?

It is common for adults who are in a relationship to have both good and bad moments. For the following questions, think about the actions of your partner and the things you may have experienced from them IN THE PAST MONTH. (Choice options: 0, 1, 2, 3, 4, 5, 6, 7, 8+ times; Refuse to answer).

- During any potential times that you may have used violence against your partner, did they ever fight back physically to defend themselves?
- Have you ever been hit or physically mistreated by your partner when you were not hitting or physically mistreating them?

**Thank you! [End of IPV Assessment – Men]**

Thank you very much for answering these questions! We really appreciate it.

Being in a relationship or married can be difficult sometimes. If you are experiencing challenges and would like advice or support, there are organisations that can help you:

- Relationship and marital support hotline: [telephone]
- Link to marital counselling services in your area: [URL]

[End of Assessment]

**The future [Women and Men]**

Thank you for responding to these questions!

1. In Jamaica for participants 16-18 years old the relationship status options were: “Single (not in a relationship)” and “In a relationship” [↑](#footnote-ref-1)
2. Following feedback received during the consultation stage with stakeholders in Jamaica during the formative evaluation, this response option was changed to “In a relationship / common-law relationship / visiting relationship” in Jamaica [↑](#footnote-ref-2)
