# Supplementary material for: A Digital Parenting Intervention With Intimate Partner Violence Prevention Content: Quantitative Pre-Post Pilot Study
Source: JMIR Form Res. 2025 Jan 3;9:e58611. doi: 10.2196/58611 (PMC11748420; doi:10.2196/58611)
Supplement: Multimedia Appendix 2 [file formative_v9i1e58611_app2.docx]

#### Multimedia appendix 2: Structure of the ParentText intervention

The parenting content of ParentText is based on the in-person Parenting for Lifelong Health programmes and was collaboratively developed with local implementing organisations and parents/caregivers in several low- and middle-income countries (LMICs), including in South Africa, Jamaica, the Philippines, Malaysia, and Sri Lanka, to ensure the material is culturally relevant. The content was translated into the local languages of the implementing countries. Topics in the programme focus on two main themes: 1) positive relationship building and 2) limit setting and nonviolent discipline. Further topics include stress reduction for parents and caregivers, child development, online child safety, sexual violence prevention, children living with disabilities, family budgeting. In addition, the chatbot also includes content on positive partner relationships and IPV prevention, based on the following five topics: (1) Treat each other as equals; (2) Become a confident parent and supportive spouse; (3) Share family responsibilities; (4) Resolve conflict peacefully; (5) Listen and talk to each other.

In line with UNICEF’s chatbot safeguarding guidelines^[[1]](#footnote-1)^, ParentText is also set up to identify high-risk keywords (for example, “ill”, “trouble”, and “fire”) in the free text field to detect potential disclosures of harm or dangerous situations. Following detection, the chatbot is designed to automatically provide the user with relevant referral contact details localised to the country. For an overview of the structure of the ParentText intervention see the table below.

| **Day** | **Morning** | **Main** | **Evening** |
| --- | --- | --- | --- |
| 1 | **IPV Baseline assessment** | Welcome |  |
| 2 | **Content: IPV Main Material** | Supportive: Activities |  |
| 3 | Supportive: Praise/Calm | Content: One on one time child | Supportive: Development |
| 4 | Supportive: Sharing | Content: Take a pause | **Check-in: IPV Topic 1 -**  **Treat each other as equals** |
| 5 | Supportive: Praise/Calm | Content: Positive instructions | Supportive: Praise/Calm |
| 6 | Content: Take a pause | Supportive: Positive instructions | **Check-in: IPV Topic 2 -**  **Become a confident parent and supportive spouse** |
| 7 | Check-in: Connection | Content: Praise | Supportive: Praise/Calm |
| 8 | Supportive: Praise/Calm | Content: Routines | **Check-in: IPV Topic 3 -**  **Share family responsibilities** |
| 9 | Supportive: Praise/Calm | Supportive: Praise/Calm | Supportive: Praise/Calm |
| 10 | Supportive: Praise/Calm | Content: Positive rules | **Check-in: IPV Topic 4 -**  **Resolve conflict peacefully** |
| 11 | Check-in: Covid | Content: Education | Supportive: Praise/Calm |
| 12 | Supportive: Praise/Calm | Content: Online | **Check-in: IPV Topic 5 -**  **Listen and talk to each other** |
| 13 | Check-in: Rules | Content: Redirection | Supportive: Share |
| 14 | Supportive: Praise/Calm | Content: Behaviour / Crying | Content: Anger management 1 |
| 15 | Check-in: Online | Content: Consequences | Check-in: On on one time |
| 16 | Supportive: Praise/Calm | Content: Ignore | Supportive: Praise/Calm |
| 17 | Supportive: Praise/Calm | Content: Emotion | Check-in: Praise |
| 18 | Check-in: Community safety | Supportive: Praise/Calm | Supportive: Praise/Calm |
| 19 | Supportive: Praise/Calm | Content: Budget with children | Supportive: Praise/Calm |
| 20 | Supportive: Praise/Calm | Supportive: Praise/Calm | Check-in: Positive |
| 21 | Check-in: Emotions | Content: Relax | Check-in: Ignore |
| 22 | Supportive: Praise/Calm | Content: Anger management 2 | Supportive: Praise/Calm |
| 23 | Supportive: Praise/Calm | Check-in: Budget with children | Content: Safety |
|  | Celebrate finishing programme | | |

*This outline is the structure of the 23-day version of the ParentText Intervention. IPV prevention content is noted in bold font. Types of messages include in the programme include Content messages, Check-in messages and Supportive messages.*

1. UNICEF. *COVID-19 Risk Communication and Community Engagement (RCCE) Guidance Note on Quantitative Data Collection*.; 2020. https://www.corecommitments.unicef.org/kp/rcce-covid19---quantitative-data-collection-guidance-note.pdf [↑](#footnote-ref-1)
