# Supplementary material for: A Digital Parenting Intervention With Intimate Partner Violence Prevention Content: Quantitative Pre-Post Pilot Study
Source: JMIR Form Res. 2025 Jan 3;9:e58611. doi: 10.2196/58611 (PMC11748420; doi:10.2196/58611)
Supplement: Multimedia Appendix 3 [file formative_v9i1e58611_app3.docx]

#### Multimedia appendix 3. Overview of ParentText IPV prevention content

A summary of the topics that are part of the IPV prevention material in ParentText is provided below. An example message corresponding with each topic is also provided. An overview of the IPV prevention content in ParentText is available in

(A full overview of the IPV prevention content in ParentText, including mother and gender-neutral versions of the material, is available in Schafer et al. 2023^a^)

| IPV Prevention Content and Example Messages in *ParentText* | | |
| --- | --- | --- |
| Topic | Content | Example message (fathers) |
| 1 | Treat each other as equals | *“Family and friends might tell you how a husband or a father should act. But both men and women benefit when they talk to each other and make decisions together. For example, next time a decision needs to be made, involve your partner, and ask what they think!”* |
| 2 | Become a confident parent and supportive spouse | *“Get involved! When fathers are engaged in parenting their children, both the child, mother, and father benefit. Set aside some time today to spend with the children.”* |
| 3 | Share family responsibilities | *“Sharing family responsibilities with your partner can make life less stressful. Think of ways you can share the workload. Doing tasks together can also make them more fun.”* |
| 4 | Resolve conflict peacefully | *“All adults have disagreements sometimes. But fighting is not an effective way to solve issues. Instead, if you start feeling angry, take a deep breath first and then respond in a calmer way.”* |
| 5 | Listen and talk to each other | *“Listening and talking to those around us are key to a more peaceful home. Talking to your partner about issues before they become bigger problems can help avoid arguments from building up.”* |

^a^ Schafer M, Lachman JM, Gardner F, Zinser P, Calderon F, Han Q, Facciola C, Clements L. Integrating intimate partner violence prevention content into a digital parenting chatbot intervention during COVID-19: Intervention development and remote data collection. BMC public health. 2023 Sep 4;23(1):1708.
