# Supplementary figures and images for: A Digital Parenting Intervention With Intimate Partner Violence Prevention Content: Quantitative Pre-Post Pilot Study
Source: JMIR Form Res. 2025 Jan 3;9:e58611. doi: 10.2196/58611 (PMC11748420; doi:10.2196/58611)

#### Multimedia appendix 4. CONSORT checklist
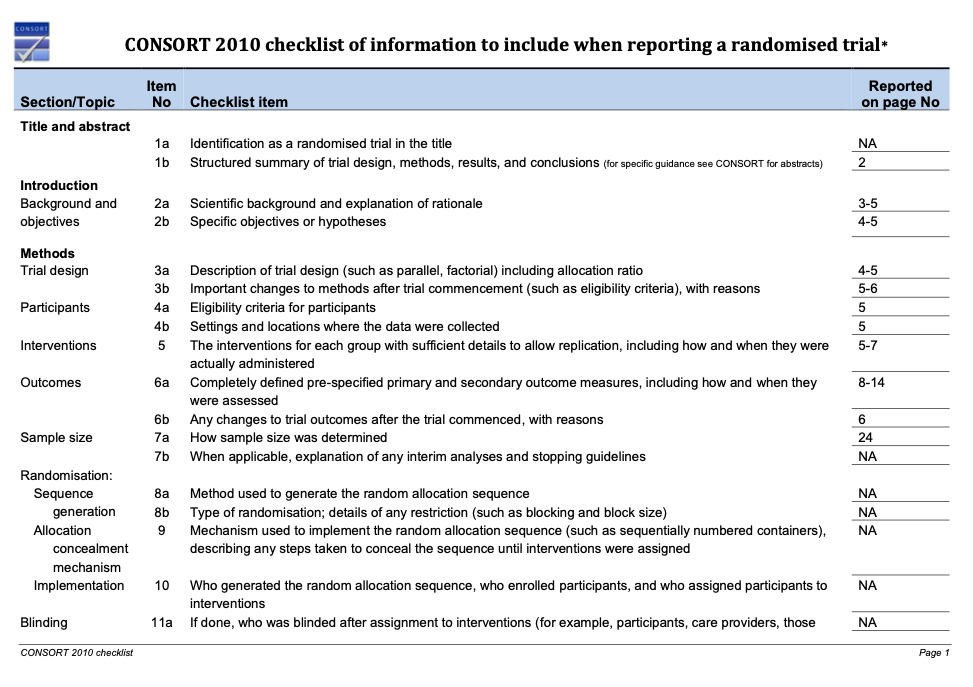


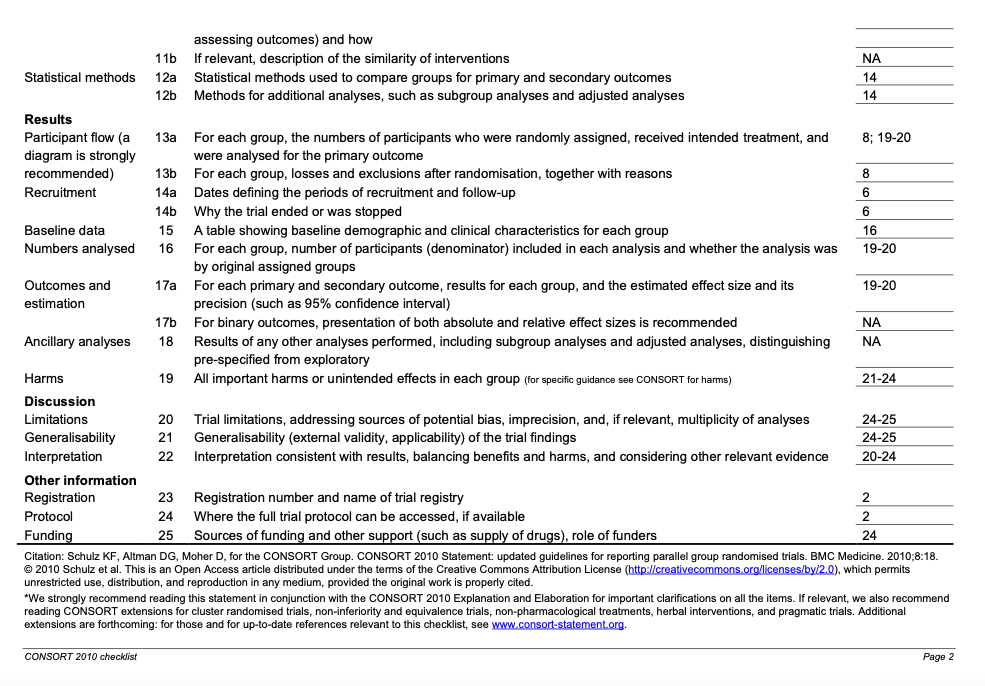

Supplement: Multimedia Appendix 4 [file formative_v9i1e58611_app4.docx]
