# Supplementary material for: A Digital Parenting Intervention With Intimate Partner Violence Prevention Content: Quantitative Pre-Post Pilot Study
Source: JMIR Form Res. 2025 Jan 3;9:e58611. doi: 10.2196/58611 (PMC11748420; doi:10.2196/58611)
Supplement: Multimedia Appendix 5 [file formative_v9i1e58611_app5.docx]

#### Multimedia appendix 5: Detailed primary and secondary outcome tables

*Additional Table 1.* Women’s past-month experience and perpetration of IPV in South Africa

|  | Baseline  (n = 19 women) | Post-test  (n = 14 women) | |  | *P* value ^b^ | |  |
| --- | --- | --- | --- | --- | --- | --- | --- |
| *Type of IPV experience ^a^* | | |  | | |  | |
| Coercive  control | 15  (78.95%) | 6  (46.15%) | | *P*=.14 | | | |
| Economic violence | 8  (42.11%) | 5  (35.71%) | | *P*=1 | | | |
| Psychological violence | 7  (36.84%) | 2  (13.33%) | | *P*=.26 | | | |
| Physical  violence | 1  (5.26%) | 1  (6.67%) | | *P*<.001 | | | |
| Sexual  violence | 1  (5.26%) | 0  (0%) | | *P*<.001 | | | |
| Overall IPV | 16  (84.21%) | 11 (73.33%) | | *P=*.01 | | | |
| *Self-dense and perpetration ^a^* | | | | | |  | |
| Self defence | 2 (10.53%) | 1 (7.14%) | | *P*=.70 | | | |
| Mistreatment | 2 (10.53%) | 0 (0%) | | *P*<.001 | | | |

^a^ % used are proportional to the baseline and post-test sample respectively

^b^ McNemar’s chi-squared test

*Additional Table 2.* Women’s past-month experience and perpetration of IPV in Jamaica

|  | Baseline  (n = 24 women) | Post-test  (n = 15 women) | |  | *P* value ^b^ | |  |
| --- | --- | --- | --- | --- | --- | --- | --- |
| *Type of IPV experience ^a^* | | |  | | |  | |
| Coercive  control | 20 (74.07%) | 12 (70.59%) | | *P=*.01 | | | |
| Economic violence | 9 (37.50%) | 3 (20%) | | *P*=.66 | | | |
| Psychological violence | 11 (44%) | 3 (17.65%) | | *P*=.69 | | | |
| Physical  Violence | 0  (0%) | 0  (0%) | | *P<*.001 | | | |
| Sexual  violence | 1  (3.70%) | 1  (5.88%) | | *P<*.001 | | | |
| Overall IPV | 24 (96%) | 13 (72.22%) | | *P*<.001 | | | |
| *Self-dense and perpetration ^a^* | | | | | |  | |
| Self defence | 0 (0%) | 0 (0%) | | *P<*.001 | | | |
| Mistreatment | 1 (3.85%) | 0 (0%) | | *P*<.001 | | | |

^a^ % used are proportional to the baseline and post-test sample respectively

^b^ McNemar’s chi-squared test

*Additional Table 3.* Men’s past-month perpetration and experience of IPV in South Africa

|  | Baseline  (n = 12 men) | Post-test  (n = 12 men) | |  | *P* value ^b^ | |  |
| --- | --- | --- | --- | --- | --- | --- | --- |
| *Type of IPV perpetration ^a^* | | |  | | |  | |
| Coercive  control | 10 (83.33%) | 9 (75%) | | *P*=.10 | | | |
| Economic violence | 9 (75%) | 4 (33.33%) | | *P*=1 | | | |
| Psychological violence | 2 (16.67%) | 3 (20%) | | *P*=.04 | | | |
| Physical  Violence | 0 (0%) | 0 (0%) | | *P<*.001 | | | |
| Sexual  Violence | 0 (0%) | 0 (0%) | | *P<*.001 | | | |
| Overall IPV | 11 (84.62%) | 9 (75%) | | *P*=.06 | | | |
| *Type of IPV experience ^a^* | | | | | |  | |
| Self defence | 0 (0%) | 0 (0%) | | *P*<.001 | | | |
| Mistreatment | 0 (0%) | 0 (0%) | | *P<*.001 | | | |

^a^ % used are proportional to the baseline and post-test sample respectively

^b^ McNemar’s chi-squared test

*Additional Table 4.* Descriptive statistics of attitudes toward gender roles and IPV among women and men in South Africa and among women in Jamaica ^a^

|  | **South Africa** | | | | | **Jamaica** | |
| --- | --- | --- | --- | --- | --- | --- | --- |
|  | *Women* | | *Men* | | | *Women* | |
|  | **Baseline**  (n = 19) | **Post-test**  (n = 14) | **Baseline**  (n = 17) | **Post-test**  (n = 16) | | **Baseline**  (n = 27) | **Post-test**  (n = 18) |
| Disagreement attitude:  A woman should obey her husband’s wishes even if she disagrees. | 2.58 (1.39) | 1.93 (1.21) | 2.82 (1.19) | | 1.94 (1.06) | 2.67 (1.30) | 2.67 (1.08) |
| Decision-making attitude:  A couple should decide together things that affect the health and well-being of the family. | 1.26 (0.45) | 2.00 (1.20) | 1.71 (0.85) | | 1.13 (0.34) | 1.21 (0.42) | 1.28 (0.46) |
| Caregiving attitude:  Fathers would benefit if they were more involved in caring for their children. | 1.42 (0.51) | 1.53 (0.52) | 1.35 (0.61) | | 1.13 (0.34) | 1.39 (0.50) | 1.39 (0.50) |
| Equality attitude:  It is natural and right that men have more power than women in the family. | 2.37 (1.12) | 2.40 (1.55) | 3.24 (1.52) | | 2.25 (1.39) | 2.79 (1.34) | 2.83 (0.99) |
| Violence attitude:  A man has a good reason to hit his wife if she disobeys him. | 1.21 (0.42) | 1.20 (0.41) | 1.53 (0.51) | | 1.13 (0.34) | 1.33 (0.68) | 1.67 (1.14) |
| Sex attitude:  It’s a wife’s obligation to have sex with her husband even if she doesn’t want to. | 1.26 (0.45) | 1.20 (0.41) | 1.88 (0.60) | | 1.31 (0.60) | 2.14 (1.27) | 2.06 (1.00) |
| Overall attitude:  Overall attitude toward gender roles and IPV | 10.11 (2.71) | 10.13 (3.89) | 11.90 (3.82) | | 8.88 (2.63) | 11.39 (3.45) | 11.89 (2.35) |

^a^ A lower score indicates more gender-equitable attitude

*Additional Table 5.* Inferential statistics of attitudes toward IPV and gender roles among women and men in South Africa and among women in Jamaica ^a^

|  | South Africa | | **Jamaica** |
| --- | --- | --- | --- |
|  | *Women  (n = 14)* | *Men*  *(n = 17)* | *Women*  *(n = 18)* |
|  | ***P* value** ^b^ | ***P* value** ^b^ | ***P* value** ^b^ |
| Disagreement attitude:  A woman should obey her husband’s wishes even if she disagrees. | *P*=.16 | *P*=.03 | *P*=1.00 |
| Decision-making attitude:  A couple should decide together things that affect the health and well-being of the family. | *P*=.04 | *P*=.02 | *P*=.64 |
| Caregiving attitude:  Fathers would benefit if they were more involved in caring for their children. | *P*=.53 | *P*=.19 | *P*=.98 |
| Equality attitude:  It is natural and right that men have more power than women in the family. | *P*=.95 | *P*=.06 | *P*=.89 |
| Violence attitude:  A man has a good reason to hit his wife if she disobeys him. | *P*=.94 | *P*=.01 | *P*=.27 |
| Sex attitude:  It’s a wife’s obligation to have sex with her husband even if she doesn’t want to. | *P*=.67 | *P*=.01 | *P*=.80 |
| Overall attitude:  Overall attitude toward gender roles and IPV | *P*=.98 | *P*=.01 | *P*=.56 |

^a^ A lower score indicates more gender-equitable attitude

^b^ *t* test

*Additional Table 6.* Descriptive statistics of past-week gender-equitable behaviours among women and men in South Africa and among women in Jamaica ^a^

|  | **South Africa** | | | | | **Jamaica** | |
| --- | --- | --- | --- | --- | --- | --- | --- |
|  | *Women* | | *Men* | | | *Women* | |
|  | **Baseline**  (n = 19) | **Post-test**  (n = 15) | **Baseline**  (n = 17) | **Post-test**  (n = 16) | | **Baseline**  (n = 26) | **Post-test**  (n = 18) |
| Feelings  How many times did you and your partner talk about your worries and feelings? | 2.00 (0.94) | 1.87 (1.30) | 1.94 (0.83) | | 2.19 (0.66) | 1.88 (0.95) | 1.83 (1.04) |
| Joint decisions  How many times did you and your partner make a decision together? | 2.16 (0.90) | 2.47 (0.83) | 2.00 (0.79) | | 2.67 (0.49) | 2.12 (1.07) | 1.83 (1.10) |
| Housework  How many times did you and your partner share housework and caregiving tasks equally? | 1.67 (0.91) | 2.21 (1.05) | 1.88 (1.11) | | 2.69 (0.48) | 1.77 (1.18) | 1.89 (1.08) |
| Respect  How many times did you explain your side of a disagreement to your partner in a respectful way? | 1.63 (1.07) | 1.53 (1.25) | 1.65 (0.93) | | 2.38 (0.81) | 1.85 (0.91) | 1.83 (0.86) |
| Overall  Overall gender-equitable behaviours | 7.37 (2.95) | 7.93 (3.53) | 7.81 (1.94) | | 9.75 (1.61) | 7.14 (3.49) | 7.39 (2.85) |

^a^ A higher score indicates more gender-equitable behaviours in the past week

*Additional Table 7*. Inferential statistics of past-week gender-equitable behaviours among women and men in South Africa and among women in Jamaica

|  | South Africa | | **Jamaica** |
| --- | --- | --- | --- |
|  | *Women  (n = 14)* | *Men*  *(n = 17)* | *Women*  *(n = 18)* |
|  | ***P* value** ^b^ | ***P* value** ^b^ | ***P* value** ^b^ |
| Feelings  How many times did you and your partner talk about your worries and feelings? | *P*=.74 | *P*=.35 | *P*=.87 |
| Joint decisions  How many times did you and your partner make a decision together? | *P*=.31 | *P*=.01 | *P*=.40 |
| Housework  How many times did you and your partner share housework and caregiving tasks equally? | *P*=.13 | *P*=.12 | *P*=.73 |
| Respect  How many times did you explain your side of a disagreement to your partner in a respectful way? | *P*=.81 | *P*=.22 | *P*=.95 |
| Overall  Overall gender-equitable behaviours | *P*=.62 | *P*=.01 | *P*=.80 |

^a^ A higher score indicates more gender-equitable behaviours in the past week

^b^ *t* test
